# *Needles, Jabs and Jags*: a qualitative exploration of barriers and facilitators to child and adult immunisation uptake among Gypsies, Travellers and Roma

**DOI:** 10.1186/s12889-017-4178-y

**Published:** 2017-03-14

**Authors:** Cath Jackson, Helen Bedford, Francine M. Cheater, Louise Condon, Carol Emslie, Lana Ireland, Philippa Kemsley, Susan Kerr, Helen J. Lewis, Julie Mytton, Karen Overend, Sarah Redsell, Zoe Richardson, Christine Shepherd, Lesley Smith, Lisa Dyson

**Affiliations:** 1 Department of Health Sciences, University of York, Heslington, York, YO10 5DD UK; 2 UCL Great Ormond Street Institute of Child Health, 30 Guilford Street, London, WC1N 1EH UK; 3 School of Health Sciences, University of East Anglia, Norwich Research Park, Norwich, NR4 7TJ UK; 4 College of Human and Health Sciences, Swansea University, Singleton Park, Swansea, Wales SA2 8PP UK; 5 Institute for Applied Health Research, School of Health & Life Sciences, Glasgow Caledonian University, Cowcaddens Road, Glasgow, G4 0BA UK; 6 University of the West of England, Centre for Child and Adolescent Health, Oakfield House, Oakfield Grove, Bristol, BS8 2BN UK; 7 Faculty of Health, Social Care and Education, Anglia Ruskin University, Young Street Site, East Road Campus, Cambridge, CB1 1PT UK; 8 York Travellers Trust, 20 Falsgrave Crescent, York, YO30 7AZ UK

**Keywords:** Immunisation, Immunization, Vaccination, Travellers, Gypsies, Roma, Showpeople, Lay beliefs, Barriers, Facilitators

## Abstract

**Background:**

Gypsies, Travellers and Roma (referred to as Travellers) are less likely to access health services including immunisation. To improve immunisation rates, it is necessary to understand what helps and hinders individuals in these communities in taking up immunisations. This study had two aims.

1. Investigate the views of Travellers in the UK on the barriers and facilitators to acceptability and uptake of immunisations and explore their ideas for improving immunisation uptake;
2. Examine whether and how these responses vary across and within communities, and for different vaccines (childhood and adult).

**Methods:**

This was a qualitative, cross-sectional interview study informed by the Social Ecological Model. Semi-structured interviews were conducted with 174 Travellers from six communities: Romanian Roma, English Gypsy/Irish Travellers (Bristol), English Gypsy (York), Romanian/Slovakian Roma, Scottish Show people (Glasgow) and Irish Traveller (London). The focus was childhood and selected adult vaccines. Data were analysed using the Framework approach.

**Results:**

Common accounts of barriers and facilitators were identified across all six Traveller communities, similar to those documented for the general population. All Roma communities experienced additional barriers of language and being in a new country. Men and women described similar barriers and facilitators although women spoke more of discrimination and low literacy. There was broad acceptance of childhood and adult immunisation across and within communities, with current parents perceived as more positive than their elders. A minority of English-speaking Travellers worried about multiple/combined childhood vaccines, adult flu and whooping cough and described barriers to booking and attending immunisation. Cultural concerns about antenatal vaccines and HPV vaccination were most evident in the Bristol English Gypsy/Irish Traveller community. Language, literacy, discrimination, poor school attendance, poverty and housing were identified as barriers across different communities. Trustful relationships with health professionals were important and continuity of care valued.

**Conclusions:**

The experience of many Travellers in this study, and the context through which they make health decisions, is changing. This large study identified key issues that should be considered when taking action to improve uptake of immunisations in Traveller families and reduce the persistent inequalities in coverage.

**Trial registration:**

Current Controlled Trials ISRCTN20019630.

**Electronic supplementary material:**

The online version of this article (doi:10.1186/s12889-017-4178-y) contains supplementary material, which is available to authorized users.

## Background

Travellers typically experience significantly poorer health and shorter life expectancy compared to the general population [1–7]. Despite this greater health need, there is low uptake of health services by Travellers, including preventive healthcare [1–6, 8]. Although there is a lack of accurate information on immunisation uptake in Traveller communities in the UK a small number of local studies using parent self-report [9–12] and NHS records [10–13] suggest low or variable uptake of childhood immunisation. Moreover there have been several well documented outbreaks of measles and whooping cough in Traveller communities [14, 15] (Note. Throughout this paper, we use the term Traveller in its broadest sense to include distinct and diverse Gypsy, Traveller and Roma communities, who may be settled or nomadic, and may live on authorised or unauthorised sites or in houses).

A large body of literature [16–23] identifies two broad categories of factors influencing uptake of childhood and adult immunisation in the general population [24–28]. The first relates to socioeconomic disadvantage where, despite vaccine acceptance, a lack of access to local and specialist services presents a barrier to uptake. The second relates to concerns about the safety or beliefs about the necessity of vaccines. There are differences in those who accept immunisation but do not complete the course (partial immunisers), those who have concerns about the safety of some vaccines but not others (selective immunisers) and those who reject immunisation altogether (non-immunisers) [29]. These diverse groups are likely to require different support and information to enable and encourage them to take up immunisation opportunities and maintain community health locally.

To date, only a few studies [9–13] have explored the barriers to immunisation uptake in Traveller communities. They identify multiple issues reflecting the difficulties in accessing wider health services experienced by marginalised, socially excluded communities [1–5, 8, 30–32]; for example a history of discrimination leading to mistrust of ‘non Traveller’ people and official institutions, poverty, low health literacy and language barriers [5]. Issues particular to immunisation include barriers to accessing primary care services (e.g. the absence of a permanent postal address for recall letters) [11], parental concerns about vaccine safety [12, 33] and objection to immunisation arising from strongly held cultural beliefs and traditions [2]. These studies are typically focused on one Traveller community and immunisation is often one component within a study exploring several health issues with Travellers limiting the extent to which the complex nature of barriers and facilitators to immunisation is explored. Whilst Traveller communities may share similar lifestyle features that distinguish them from the general population, beliefs and cultural traditions can vary [34]. It is, therefore important to understand whether, and how, factors that promote or inhibit immunisation differ among specific communities. Moreover, barriers may be specific to particular vaccines e.g. measles, mumps and rubella (MMR) vaccine or differ for adult and childhood vaccines. Issues associated with newer vaccines, for example, childhood flu have not always been considered, nor have evolving views about previously controversial vaccines (e.g. whooping cough, MMR) or the views of more recent migrant communities in the UK e.g. Romanian and Slovakian Roma. The UNITING study set out to advance understanding by addressing the limitations of previous research. It was a three-phase qualitative study: interviews with Travellers in the UK, interviews with Service Providers followed by workshops with Travellers and Service Providers to prioritise interventions for increasing immunisation uptake. In this paper we present Phase 1, interviews with Travellers. The aims were as follows:

1. Investigate the views of Travellers in the UK on the barriers and facilitators to acceptability and uptake of immunisations and explore their ideas for improving immunisation uptake;
2. Examine whether and how these responses vary across and within communities, and for different vaccines (childhood and adult).

## Methods

The methods of the three study phases are described elsewhere [35]. The theoretical framework underpinning the study was the Social Ecological Model (SEM) [36] which recognises that the determinants of individuals’ behaviour are complex, multifaceted and operate at a number of levels (intrapersonal, interpersonal, institutional, community, policy). We used the SEM to ensure that all levels of potential influence on immunisation behaviours were explored. Acknowledging the multi-level influences on immunisation uptake is particularly relevant for understanding health behaviours in socially excluded communities such as Travellers and for informing future interventions for both policy and practice.

### Setting and participants

The research focused on six Traveller communities based in four UK cities (see Table 1). The English Gypsy, European Roma and Irish Traveller communities are recognised legally as ethnic minorities [37, 38]; despite different beliefs, customs and languages, they share common features of lifestyle and culture [39] and are genealogically and linguistically related [40]. In contrast the Scottish Showpeople (travelling show, circus and fairground families) are not recognised as part of the “traditional Travellers” ethnic group. Indeed, it is reported that this group does not want to have recognised ethnic minority status, self-defining as business/cultural communities. It is only their traditionally nomadic lifestyle that means that legally they are labelled as Travellers [41]. Further detail on the six Traveller communities is presented elsewhere [35, 42].

**Table 1:** Demographic characteristics of participants

	All	Bristol Romanian Roma	Bristol English Gypsy/Irish Traveller		York English Gypsy	Glasgow Roma		Glasgow Scottish Showpeople^d^	London Irish Traveller
			English Gypsy^a^	Irish Traveller^b^		Romanian Roma	Slovakian Roma^c^		
Total	174	24	15	9	48	17	20	14	27
Used Interpreter	47	19	0	0	0	12	16	0	0
Gender									
Female	139	14	10	7	37	17	17	10	27
Male	35	10	5	2	11	0	3	4	0
Family role									
Mother	64	9	5	4	19	8	7	5	7
Grandmother	33	3	4	1	6	3	5	3	8
Pregnant woman	5	0	0	0	2	0	0	0	3
Woman no children	8	0	1	1	3	0	0	1	2
Adolescent girl with children	5	2	0	0	0	1	1	1	0
Adolescent girl no children	24	0	0	1	7	4	5	0	7
Father	19	6	2	2	5	0	2	2	0
Grandfather	5	1	0	0	2	0	0	2	0
Male no children	11	3	3	0	4	0	1	0	0
Housing									
House/Flat	112	23	0	6	24	17	20	3	19
Authorised site - caravan/trailer	45	0	11	3	23	0	0	0	8
Authorised site - chalet	15	0	4	0	0	0	0	11	0
Bed and Breakfast	1	1	0	0	0	0	0	0	0
Missing	1	0	0	0	1	0	0	0	0
Self-reported Immunisation status of participants									
Full	59	7	4	2	24	5	4	11	2
Partial	40	5	7	2	7	4	4	3	8
None	11	1	0	3	4	1	0	0	2
Missing	64	11	4	2	13	7	12	0	15
Self-reported Immunisation status of participants’ children									
Full	69	10	8	6	20	7	10	0	8
Partial	17	4	0	1	2	0	2	0	8
None	2	0	2	0	0	0	0	0	0
N/A	44	3	4	2	16	5	4	1	9
Missing	42	7	1	0	10	5	4	13	2

*Note*: ^a^One participant is a Welsh Gypsy. ^b^One participant has married into the Irish Traveller community. ^c^One participant is Hungarian. ^d^One participant has married into the Scottish Showpeople community

Within each Traveller community we set out to recruit men (approximately a quarter of the sample) and women living in extended families across generations. We included young women planning families, parents and grandparents to capture a life span/cross-generational perspective as well as adolescents eligible for their three-in-one booster (diphtheria, tetanus, poliomyelitis, given at 13–18 years), girls eligible for HPV vaccine (given at 12–13 years in school); and adults eligible for the flu vaccine (pregnant women, over 65 years and those with specified long term conditions) and whooping cough vaccine (pregnant women). We also sought to include a mix of full immunisers/partial immuniser and non-immunisers (based on self-report). We planned to interview approximately 22–32 participants in each community, enabling us to look for potential differences and similarities in views *within* a community as well as draw out meaningful comparisons *across* communities.

### Access and recruitment

Access to potential participants was enabled by gatekeepers who had longstanding relationships with the communities. These gatekeepers initially spoke with Travellers about the study, and distributed printed information sheets for them to take away and discuss. These documents had been developed for each Traveller community through public involvement with members of the local community, and were translated for the Roma communities. The gatekeepers identified potential participants for the study and usually facilitated interview scheduling for the local research teams. Snowball sampling [43] also occurred. Participants were given a £15 gift voucher to thank them for their time. Recruitment and data collection occurred between December 2013 and April 2015.

### Data collection

A mixture of one-to-one and small group interviews, depending on participant preference, with members of the same family/peer group were conducted. Interviews were held in locations known to participants, for example at home or in a community centre. Almost all interviews with the Roma participants were conducted with the assistance of an interpreter. With the consent of participants, interviews were recorded digitally.

A topic guide was developed to ensure consistency of data collection both within and across the six communities although the format was flexible to allow participants to raise additional issues they considered important. We focused primarily on issues arising from the UK childhood immunisation schedule [44] but also explored views on antenatal whooping cough and flu vaccine in pregnancy as well as in older and at risk adults. Participants were asked at the start of the interview which word they used for vaccinations and this was used throughout. The terms injections, needles, jabs, jags, immunisations, inoculations, vaccinations and vaccines were identified. The researcher then asked participants for a story about any experience of having a vaccination, their views on having injections and perceived views of others, their immunisation experiences (for self, children) and ideas for increasing take up of vaccinations. Throughout the interview participants were prompted to consider the influence of the five levels of the SEM (described to participants as: self, family/friends, community, health professionals, local/national policy makers) on their views, experiences and ideas.

### Data analysis

The analysis was led by the research team in York. Research team members in the other three cities were involved at different stages to enhance rigour and to ensure that the local context in which the data were collected was retained. A data analysis protocol was developed to ensure consistency across the team.

#### Within-community analysis

The interviews were transcribed verbatim and data subjected to thematic analysis using the Framework approach [45] which is designed to address policy-related questions. Transcripts were checked for accuracy against the audio-recording. Ten percent of the transcripts of interviews with Roma participants, selected at random, were checked against the audio-recording by an independent interpreter.

The stages of Framework analysis were undertaken independently for each Traveller community. Participant-based group analysis [46] was used to analyse the group interviews, with the contribution of each individual within the interview being analysed separately. QSR NVivo 10 and Microsoft Excel 2010 software packages facilitated data management.

##### Familiarisation

The researchers in York read all of the interview transcripts from York and Bristol (the first data collection sites) to record emerging ideas and recurrent themes that were relevant to the aims of the study.

##### Constructing a thematic framework

A thematic framework was developed using 16 interview transcripts from York and Bristol which were selected to reflect a mix of participants. The framework (see Additional file 1) was organised by the emergent ideas and themes (identified in the previous stage). A decision was taken at the outset to embed the SEM into the interview topic guide, as such the data collected reflected the different levels of influence but were not forced into a pre-specified theoretical framework which might have constrained the findings. The framework was applied to a further four transcripts by a second researcher and refined when necessary.

##### Indexing and charting

The thematic framework was systematically applied to the interview data from across all four cities. Charts were produced in NVivo for each theme and summaries of responses from participants and verbatim quotes were entered. A sub sample of the completed charts for each Traveller community was reviewed by a different researcher to check the detail and sufficiency of the summaries and quotes.

##### Mapping and Interpretation

The completed charts were exported from NVivo into Excel. These were reviewed and interrogated to compare and contrast views, seek patterns, connections and explanations within the data. Descriptive Findings documents were written for each Traveller community focusing on the barriers and facilitators to uptake of immunisation. The five levels of influence from the SEM were evident within the barriers and facilitators. The local research teams in each city then reviewed their documents to: (1) check that the interpretation of the local data by the analysis team reflected the intended meaning during the interviews and (2) where necessary, provide local context to assist interpretation.

#### Cross-community synthesis

The final step was a thematic cross-community synthesis that took account of the inferences derived from all the interview data for the Traveller sample as a whole [47]. Using the Descriptive Findings documents and charts for each Traveller community, the data across all six communities were synthesised by four researchers to explore similarities and differences in views on barriers and facilitators to immunisation. The final themes and sub-themes were mapped to the five levels of influence within the SEM. This final level of analysis was reviewed by the entire research team.

## Results

### Participants

We interviewed 174 Travellers in total. Thirty eight Travellers participated in individual interviews; the remainder in group interviews. The demographic characteristics of the participants are presented in Table 1. We achieved a mix of gender and generations, as intended. We also achieved a mix of self-reported immunisation status although data were missing from over a third of participants. Most participants lived on an authorised caravan, trailer or chalet site or were housed. We did not recruit anyone currently living on the roadside or on unauthorised encampments.

### Barriers and facilitators to uptake of immunisation

The five broad themes and their corresponding sub-themes that emerged from the thematic framework for the six Traveller communities are presented in Table 2 and described below. Where there were differences in views by community, gender or for specific vaccines these are highlighted. Many of the themes/sub-themes were considered to be relevant to more than one level of the SEM (see Table 2). Less interview data emerged that mapped onto the policy level compared to the other four levels (intra-personal, inter-personal, institutional and community).

**Table 2:** Themes, sub-themes and corresponding level of SEM

Theme	Sub-theme	Level of SEM
Vaccine knowledge	Levels of knowledge	Intra-personal
	Sources of information and advice	Intra-personal, Inter-personal, Institutional, Community
Acceptance of immunisation	Reasons for acceptance of immunisation	Intra-personal, Policy
	Reasons for concerns about immunisation	Intra-personal, Inter-personal, Community
	Beliefs about specific vaccines	Intra-personal, Inter-personal, Community
	Inter-generational change in beliefs about immunisation	Intra-personal, Inter-personal, Institutional, Community
	Decision-making about immunisation	Intra-personal, Inter-persona
Socio-cultural factors	Language and literacy	Inter-personal, Institutional, Community
	Discrimination	Institutional
	Housing	Institutional
	Attendance at school	Intra-personal, Institutional, Community
	Travelling	Institutional, Community
Accessible health services	Relationships with health professionals	Institutional
	Recall and reminders	Institutional
	Attending appointments	Institutional
National strategies	Payment and incentives	Policy

#### Vaccine knowledge

##### Levels of knowledge

There was widespread understanding across all six communities that immunisation protects against diseases and prevents infection spreading. A minority had good understanding of the schedule for childhood vaccinations and how vaccines work, although there was little reference to the concept of ‘herd immunity’. The common perception among the English-speaking communities was that the current generation of parents of young children are more knowledgeable than previous generations due to better literacy.

**LT001a, Irish Traveller, Mother, London:** *A lot of the Travelling community like you saw today are starting to read and write so they’ll be able to look and read the leaflets properly… I think it’s the old people they don’t really understand what injections are for because they probably didn’t get their kids done.*

Knowledge of specific immunisations was more variable. Most of the Romanian Roma participants in Bristol and Glasgow appeared to have limited understanding of specific vaccines, the diseases they protect against and the time at which they are routinely provided. However some Slovakian Roma participants in Glasgow were more knowledgeable. Awareness of the childhood whooping cough and MMR vaccinations was particularly evident in the English-speaking Traveller communities, seemingly because of the controversies surrounding their safety in past decades. There was also a good level of awareness of the existence of the HPV vaccination amongst young women and their mothers with the exception of adolescent girls from the London Irish Traveller community. A minority of participants in the York English Gypsy, Bristol and Glasgow Roma communities were unclear about the type of cancer the HPV vaccine protected against, or believed it prevented all cancers. Across all six communities there was infrequent reference to more recently introduced childhood vaccinations (e.g. rotavirus, childhood flu). Knowledge of adult vaccinations (with the exception of flu) tended to be less than for childhood vaccinations; in particular the Bristol and Glasgow Roma participants appeared less familiar with the availability and purpose of adult vaccinations.

**BT202, Romanian Roma, Father, Bristol (via his wife who was translating on his behalf):** *He says he has not heard of immunisation for adults which is why he was surprised when his brother said ‘he has done one’ … he knew about vaccinations for children but not for adults.*

##### Sources of information and advice

Travellers in all communities overwhelmingly identified health professionals as their key sources of verbal and written information about both childhood (GP, Health Visitor, Midwife, Support Worker) and adult (GP, Practice Nurse, Midwife) immunisation because of their training.

**YT007a, English Gypsy, Mother, York:** *The information you need, for whichever injection you are getting, the doctors will provide you with… anyway so you wouldn’t need to go and have a look anywhere else would you?*

The other important information source was family and community. Whilst Travellers across all communities spoke of a shift from family towards health professionals as the primary source of health information, the sharing of experiential knowledge and advice was still passed down via ‘*word of mouth*’, especially amongst Irish Travellers in Bristol and London. This occurred through intergenerational relations (grandmothers, mothers and children) and certain female community members who, as a result of their greater experience and knowledge of vaccinations, served as immunisation ‘*advocates*’, providing information and advice to others.

A small number of mothers and adolescent girls across all six communities described receiving invitation letters and useful information about relevant immunisations (i.e. flu, HPV and teenage booster) from schools. Views on the usefulness of information gained through the media, social media and the Internet (e.g. Google, YouTube) were mixed across all communities, perceived by some as biased or ‘*scare mongering*’ and by others as accurate, balanced sources. Several spoke of using it to make sense of advice provided by health professionals, checking out side effects of vaccinations, symptoms of diseases and translating immunisation information. A few participants observed that it is the ‘*young people*’ who mainly use the Internet and will often help their parents who do not have the IT or literacy skills to access information in this way.

**Interviewer:** *Do you Google things as well?*

**BT109a, English Gypsy, Mother, Bristol:** *No, my love, I can’t even use a computer……me daughter does it all.*

Facebook was the most commonly discussed form of social media. In the London Irish Traveller and Bristol English Gypsy/Irish Traveller communities Facebook was considered a good method of disseminating information as it was used by people of all ages in their community. However, for other participants across the English-speaking communities the use of social media was considered an inappropriate medium to share information about vaccination because the subject was viewed as a private family matter. More relevant to the Roma communities was having no access to the Internet. Women from the Scottish Showpeople noticeably discussed the negative or biased portrayal of information in the media/on the Internet about MMR specifically, for example, coverage of Tony Blair’s refusal to reveal whether his son had had MMR, Andrew Wakefield’s Facebook page and other Facebook pages about the MMR-Autism link.

#### Acceptance of immunisation

Although there were some subtle differences across communities, most Travellers’ accounts revealed beliefs that the protective benefits of immunisation outweighed the risks such as minor side effects or short-lived discomfort and upset, leading to immunisation take up for themselves and their children. Immunisation was viewed as a ‘*normal*’ thing to do. This view was expressed by almost all of the Bristol and Glasgow Roma, three quarters of the Bristol English Gypsy/Irish Traveller, Scottish Showpeople and half of the York English Gypsy and London Irish Traveller communities.

**YT006b, English Gypsy, Mother, York:** *It’s not nice putting them through it, but I guess they need it don’t they, so that they don’t catch anything. I know if I didn’t take her and then anything did happen what could have protected her … I think she’d be worse off.*

**BT112b, Irish Traveller, Mother, Bristol:** *I think for us it’s natural to get injections for the babies, it’s natural, it’s what you do… it’s all part of rearing ‘em up and keeping ‘em safe innit?*

Others, particularly amongst the York English Gypsy and London Irish Traveller communities, expressed more mixed feelings. Discomfort to the child of having the injection, ‘*contamination*’ from needles, potential side effects, not always believing that vaccines work and uncertainty about what they contain were concerns raised. Rather than uncritically accepting vaccinations, these participants described weighing up advantages and disadvantages before usually deciding to go ahead, concluding that the diseases vaccinations protect against pose greater risks than the vaccines themselves.

**LT007a, Irish Traveller, Mother, London:** *Yeah I mean if it, if you’re told this is gonna protect your child against something that is potentially fatal then obviously you’re gonna do it. But then …you’ve got…, “Oh but something could be wrong with your baby if you give her that”, do you know what I mean? It’s a lot to weigh up that. Obviously I gave mine them and, touch wood, mine are all fine.*

##### Reasons for acceptance of immunisation

Across every community, irrespective of level of immunisation acceptance, the desire to’*do the best for your children*’ emerged strongly in Travellers’ accounts, with one English Gypsy mother from Bristol describing Travellers as superstitious and very concerned about their children’s health.

**BT109a, English Gypsy, Mother, Bristol:** [Travellers are] *very superstitious and very funny about their children… overly protective. In a Gypsy’s eyes there’s no other child like your own.*

Another reason to vaccinate amongst older participants from the English-speaking communities was personal experience (or seeing others’ experience) of preventable diseases, particularly the consequences of measles, whooping cough, meningitis and cervical cancer.

**LT005a, Irish Traveller, Grandmother, London:** *I remember my nieces and nephews used to get… Whooping Cough, and you’d never hear about any vaccination for it, it’s frightening ‘cos they keeps coughing and they go blue coughing the whole time… And my child had … measles at that time. I had to keep him in, in the caravan but I had to put him into darkness… it was my mother used to be telling me, keep him in darkness, don’t leave him out in the light, and get Calpol or whatever you can get for him…he was about 2 weeks like that.*

Personal experience or seeing no ill effects for a first child post-vaccination also encouraged some Travellers to have subsequent children immunised. Within the London Irish Traveller community this was seen as particularly relevant to current acceptance of MMR. Other reasons for proceeding with vaccination were offered by Roma participants. In Glasgow, some Roma Travellers had been asked if they were up-to-date with their vaccinations when applying for work and believed that their children would not be allowed to attend school unless they had all their childhood vaccinations. Two Bristol Roma participants described viewing vaccinations as a way to integrate into UK society, given that this is the accepted norm here.

##### Reasons for concerns about immunisation

Concerns about ‘*overload*’ to the immune system from multiple or combined childhood vaccines in general and more specifically about MMR were mentioned by a small minority in each of the English-speaking Traveller communities, and by one Bristol Roma mother. In some instances families in the Scottish Showpeople, Bristol Irish Traveller/English Gypsy and York English Gypsy communities had paid privately for single measles, mumps and rubella vaccines instead of accepting the combined MMR vaccine for their children.

**GT202a, Scottish Showperson, Mother, Glasgow:** *You’ve worried for months about getting jags and then it takes 2 seconds for that virus in your child and it’s in it canna come out. It’s horrible, it’s not nice to think about. It’s the three live viruses in the one needle… too much.*

A similarly small minority of participants (sometimes just one person) in the English Gypsy and Irish Traveller communities questioned the value of immunisation because someone they were aware of in their community or their own family was considered to have been seriously harmed by a vaccination. Noticeably almost half of the Scottish Showpeople women voiced concerns about MMR. This appeared to be a consequence of two mothers in their community having a child with autism, one of whom believed the MMR vaccination to be the cause.

Only three Travellers in the sample as a whole talked about completely rejecting immunisation. A Bristol Irish Traveller explained she had not had her children vaccinated because vaccines contain too many chemicals, preferring to look after her children with pain killers and calamine lotion for chicken pox, as her own mother had done for her children. In York, a mother said that she did not believe in the value of injections, describing them as ‘*parasites*’ that cause brain damage. Her unvaccinated daughter commented that she trusted her own mother’s judgement about immunisation and that she did not intend to have her own children vaccinated in the future.

##### Beliefs about specific vaccines

Concerns were raised about specific vaccines across all six communities. These largely related to MMR, whooping cough, HPV and adult flu. As mentioned above MMR was discussed extensively by women in the Scottish Showpeople community where unease about this vaccination was greater than in the other Traveller communities. In contrast, in Bristol, York and London previous measles outbreaks in their communities were considered to have served to increase acceptance of MMR among most families in these communities.

The whooping cough vaccine in pregnancy was a particular issue for the Bristol English Gypsy/Irish Travellers with more than half of the women interviewed stating that they did not or would not have it when pregnant. Concerns seemed to stem from a common belief, expressed by both men and women, that the ‘*immune system is low*’ in pregnancy and that injections should be given after the baby is born. There was also a strong, long held community belief amongst older Travellers that the whooping cough vaccine leads to brain damage and disability.

**BT108d, Irish Traveller, Father, Bristol:** *These* [whooping cough] *are needles that the women don’t take when they are pregnant because to them it’s God’s fate, you just don’t inject when a woman’s having a baby …you just leave it alone and leave it in God’s hands. What will be will be.*

Safety concerns about HPV were mentioned by a few women who had read media stories of serious side effects but the more common issue raised by a minority of mothers and fathers/grandfathers in the English Gypsy and Irish Traveller communities, related to the belief that having the vaccination would imply that Traveller girls were promiscuous. Within these accounts, HPV elicited strong views with respect to the moral overtones of accepting the HPV vaccination and how their community, in which no sex before marriage and a partner for life were powerfully held beliefs, would view this.

**LT008b: Irish Traveller, Mother, London.** *There’s a new one we are all a bit wary about, the HPV for the young ones. And our young ones, they’re clean when they get married so we don’t, we’re not into than kinda giving that one to the young ones. …our girls aren’t promiscuous, look after the girls’ reputations do you know what I mean?*

These views were counterbalanced by Traveller participants from other communities, particularly mothers, their adolescent daughters and grandmothers, who were positively predisposed to the HPV vaccination as a preventive measure for cancer.

Concerns about becoming ill after the adult flu vaccination were raised by a minority across all the English-speaking communities. This vaccination appeared to be declined on the basis that flu was less important than other vaccine preventable diseases, or a belief that having the vaccine gave the person flu (based on personal experience or observations of others who had received the vaccination).

**BT108a, Married to an Irish Traveller, Father, Bristol:** *When I went and had it* [flu vaccination] *I was bad for days after the needle, but I haven’t had it since…it put me off having it to be honest.*

##### Inter-generational change in beliefs about immunisation

Travellers’ accounts referred to an inter-generational shift in beliefs about immunisation with the current generation of parents tending to be more positive than their grandparents, and sometimes their parents. There was reference to *‘old people’s stories’* and the traditional view of using natural remedies to cure illness as well as Travellers being frightened of immunisation in the past. This change from immunisation being viewed as dangerous to viewing vaccinations as a protective measure was mentioned by some Travellers in every community, with the exception of the Scottish Showpeople.

A number of reasons were offered for this change in beliefs. It was suggested that Traveller communities are now more integrated into society because many are more settled and are influenced by their associations with non-Travellers through working locally and their children attending local schools. This may have led to increased confidence to access and trust health services. Improved literacy was also identified with parents now proactively accessing information about immunisation from a variety of sources and relying less on ‘*passed down*’ knowledge through grandparents and mothers to daughters.

**BT218a, Romanian Roma, Father, Bristol:** *I think our generation, up to about 30, 35 years old, we accept the idea of immunisation. The older ones… they are a bit* [uncertain]*… because they didn’t go to the doctor so often.*

##### Decision-making about Immunisation

With the exception of Bristol Roma who did not volunteer information on this issue, many mothers considered they were the main decision-maker about childhood vaccination. This was considered the norm within each community principally because mothers were viewed as having the main responsibility for bringing up their children.

**Interviewer:** *Do you ever consult your husband on health things and health decisions? I mean if, if you were unsure about a vaccine, like the MMR we’ve talked about, would you say, you know, to your husband?*

**LT010b Irish Traveller, Grandmother, London :** *No, I mean, no, I mean… No, he mainly leaves everything to me.*

Nevertheless, other mothers described making the decision with or seeking agreement from their partners. In the York English Gypsy and Glasgow Scottish Showpeople communities most of the men concurred with the view that their female partner knows more about childhood immunisation than they do. They described immunisation decision-making as ‘*more a woman’s thing*’ (**YT013a, English Gypsy, Father, York**).

#### Socio-cultural factors

##### Language and literacy

Significant language and literacy barriers existed for the Bristol and Glasgow Roma communities leading to a strong reliance on interpreters who were in short supply. Challenges were identified in terms of being able to communicate with health professionals within consultations as well as understanding written information and invitation letters for immunisation.

**GT102a, Slovakian Roma, Mother, Glasgow:** *I took my son twice* [for vaccinations]*. I didn’t know what they were actually saying, I didn’t know what it was for; I didn’t understood. If I go somewhere I do manage to make myself understood, that time I didn’t.*

Literacy was also seen as a barrier among the English-speaking communities in York and Bristol; this was not exclusively related to older community members. Of those who discussed literacy, many described people being unable to read immunisation leaflets or letters/texts about appointments as well as struggling to make sense of conversations with health professionals, particularly if GPs use medical jargon.

**BT112a, Irish Traveller, Mother, Bristol:** *She’s good, I like her* [the Health Visitor]*. You’re worried about things and you say to her like “I don’t know what, what I should do”. She, she’ll tell you, but she’ll tell you in our words that we understand… whereas if you go to a doctor… you’ll sit there and you’re thinking “I don’t know what you’re saying but I’ll pretend I know otherwise I’ll look stupid”, you know what I mean?*

Those Travellers who spoke of their own difficulty with reading and writing described how they rely on family and other community members for information about immunisation, to read out letters about appointments and to accompany them to consultations with health professionals. A strong issue to emerge was a preference for spoken information. Translating written information was seen as only a partial solution for Roma families as it did not address literacy difficulties. Scottish Showpeople in Glasgow and Irish Travellers in London did not mention their own literacy levels or those of their family and wider community.

##### Discrimination

A minority of female participants from the English-speaking Traveller communities (sometimes just one person) described feeling discriminated and marginalised from health services. This included discrimination by health professionals and support staff in NHS premises. These women believed their poor health service encounters to be because they are perceived as ‘*thieves, vagabonds, unhygienic who would steal children and all get tarred with the same brush*’ **(GT208a, Scottish Showperson, Grandmother, Glasgow)**. In contrast, no Roma participants in Bristol or Glasgow described experiencing discrimination from health services. Indeed two Slovakian Roma, a grandmother and a father, commented that they were treated more kindly in Scotland, ‘*normally, like the others*’ (**GT103d, Slovakian Roma, Grandmother, Glasgow**) than in Slovakia where racism towards Roma was referred to as a problem.

##### Housing

Only two Traveller participants, one Bristol Roma Father and a York English Gypsy Mother, specifically spoke of how their housing (one on an official site, one in a house) facilitated take up of immunisations because their families are more integrated into society and are located close to the local GP practice. Conversely a small number of Scottish Showpeople commented that their immunisation invitation/recall letters sometimes get lost because of the communal post box used on site.

##### Attendance at school

A small number of female Travellers discussed how some adolescent girls are home educated and therefore not attending secondary school which can present a barrier to school-based immunisations such as HPV. This was evident across all Traveller communities with the exception of the Glasgow Scottish Showpeople where school attendance was described as good. A number of reasons were offered for this non-attendance. There was a view from some York English Gypsy participants that some girls are withdrawn from school as they enter puberty because their fathers do not like them mixing with non-Traveller boys. A minority of Slovakian Roma adolescents in Glasgow reported experiencing racism and discrimination at school.

**GT111a, Slovakian Roma, Adolescent girl with no children, Glasgow**: *I never have been called a Gypsy, just in the school once… They don’t know anything about you and they’re just gonna call you like that…You don’t want to talk to anyone now… it feels like you’re different from them, like you have different, like everything different, not like them.*

Whilst some London Irish Traveller mothers said their daughters had missed HPV as they were travelling or were not in school that day. One spoke of how she struggled to access the HPV injection for her home-schooled daughter.

##### Travelling

Many York English Gypsy and Scottish Showpeople explained that they were mainly settled now, travelling only in the summer months to fairs. This had facilitated uptake of immunisation because they routinely access GP services and book immunisation appointments around travelling commitments.

**GT201b, Scottish Showperson, Mother, Glasgow:** *If you was offered a jag and you wasn’t here and you was out travelling, you would probably make another appointment wouldn’t you. You wouldn’t miss it. If you wanted it [immunisation] you wouldn’t miss it.*

Within the Bristol English Gypsy/Irish Traveller and London Irish Travellers, views on the impact of travelling on immunisation uptake were more mixed. In Bristol an English Gypsy mother and an Irish Traveller grandmother spoke about how travelling makes it difficult to get to appointments for children’s vaccinations, because invitation letters are not received or appointments made when away and therefore it becomes a *‘hassle’* to attend.

**BT111b, English Gypsy, Mother, Bristol:** *When Travellers have to book things, they never keep to it if they’re travelling about… We think injections are good but they just can’t be arsed with the hassle …cos they’re never in one place more than, well, a couple of weeks … cos the doctor won’t see you half the time, will he?*

The Roma participants did not discuss travelling, except in the context of arriving in the UK.

#### Accessible health services

A minority of Travellers across all six communities described problems accessing health services. Issues raised included the difficulty of registering with a GP practice without a fixed address or living on an unofficial site, frustration in getting through to the GP practice by phone to book an appointment, and being unable to get an appointment quickly — with some reporting that they had to wait for up to two weeks for an appointment, a particular problem for those who are travelling.

**LT014b, Irish Traveller, Mother, London:** *It’s very hard to get an appointment innit?*

**LT014a, Irish Traveller, Mother, London:** *Yeah, it is hard. They might give you an appointment for 2 weeks’ time, by 2 weeks’ time I’m forgetting about it anyway.*

One consequence of these frustrations was that some Travellers would use out of hours doctors or A&E for general healthcare which some described as providing a better service because they were seen quicker, the ‘*doctor looks at you more carefully*’ and it involves less paperwork for those who struggle to fill in forms.

##### Relationships with health professionals

The importance of relationships with health professionals, with GPs and Health Visitors particularly, emerged strongly across all six communities. Many Travellers, predominantly women, described positive relationships based on trust and respect that often developed by attending the same GP practice and seeing the same health professionals over a prolonged period of time.

**YT002a, English Gypsy, Mother, York:** *It’s the same practice so we know the Doctors and I really wouldn’t want to move myself or my kids from them because they know us as if you’re equal, if you know what I mean.* [I’m] *not just a patient, they know our history and get on with them.*

Other positive features that were mentioned were regular contact by GP practices to remind families to attend appointments (seen as evidence that they care); and confidence to telephone the GP practice to ask about anything they had concerns about.

Specific to immunisation, positive examples of relationships with health professionals included a GP who had encouraged a distressed child to sit still to have an injection; and Practice Nurses and Health Visitors taking time to discuss with parents the importance of having a particular immunisation. A small number of English Gypsy Travellers in York, Irish Travellers in London and Glasgow Scottish Showpeople preferred to have their vaccinations done by a GP in the practice rather than by an outreach Health Visitor, a School Nurse or a Pharmacist. This appeared to be based on either having a more established relationship with the GP or seeing the GP as having greater expertise and authority.

There were a few accounts of negative encounters with health professionals which had damaged relationships. For example several London Irish Travellers offered examples of when Health Visitors had not taken time to discuss immunisations fully and an English Gypsy grandmother spoke of how in the past a GP had called her an uncaring parent when she would not have her children immunised because she was concerned about vaccine safety.

**BT105a, English Gypsy, Grandmother, Bristol:** *It was only out of care that we were doing it, it wasn’t that you wanted to neglect them.*

In contrast, the Roma participants in Bristol and Glasgow did not identify any negative experiences with health professionals; and only two participants from the Scottish Show people referred to poor experiences.

##### Recall and reminders

Traveller participants, particularly women, across all six communities, reported a range of methods by which they are prompted to attend for immunisations for their children or themselves. Most commonly they referred to letters from their GP practice or from school to inform them that a vaccination is due. Some spoke about receiving texts and telephone calls as a reminder to attend or to rebook a missed appointment. These recall and reminder systems appeared to be seen as effective for the majority of people including those who travel (when texts are useful). Those with literacy and language barriers employed a variety of strategies to navigate these systems, including using Google Translate to understand the letter and asking staff at the GP practice to read out the letter.

**YT003a, English Gypsy, Mother, York:** *Because like the dates on the letter and which we couldn’t read because I wasn’t attending these* [literacy] *classes then so I took the letter to the receptionist and she read it out, have a seat until the nurse calls you through, she called us through we went through and she read the letter out and she says, “Oh it’s just because you are asthmatic and this is to like stop you getting infections and that from the weather”.*

Several Traveller women in every community also referred to being reminded about immunisations through home visits from Midwives, Health Visitors and bi-lingual Support Workers, their red books (Personal Child Health Records) and attending the GP practice for other reasons (e.g. getting a child weighed). Small numbers of men and women described how when they attended for appointments for blood tests and check-up appointments for long term conditions health professionals took the opportunity to check whether their immunisations were due. These face-to-face reminders appeared to be particularly well received offering the opportunity for an explanation of the vaccinations to be provided as well as overcoming language and literacy barriers.

##### Attending appointments

Most participants, the Glasgow Scottish Showpeople participants particularly, did not appear to have problems attending appointments for immunisations.

**BT101a, English Gypsy, Mother, Bristol:** *Just take your child to the doctors, wait for your name to be shout, take ‘em in, and within seconds she’d be jabbed, out, that’s it, all over with.*

Only two Traveller participants (both Bristol Irish Travellers) suggested that it is not usual for Travellers to follow appointments within their culture.

**BT106a, Irish Traveller, Grandmother, Bristol:** *Travellers don’t like working on appointments because they’re either moving away or the timing is wrong or they haven’t got a way of going.*

Drop-in or walk-in clinics for immunisations were seen as a sensible approach to free up appointments in GP practices, avoid people having to wait two weeks for an appointment and for groups of mothers to share transport. They were seen as particularly convenient for those who struggle to get to the GP practice for pre-booked appointments, for example mothers with many children, people working long, anti-social hours and those who regularly travel.

There were mixed views on the value of outreach. Within the York English Traveller community the general perception appeared to be that whilst visits from Health Visitors and Midwives provided a good opportunity to discuss immunisations they should only be administering vaccinations to those who struggle to attend the GP practice, for example older people.

**YT005a, English Gypsy, Grandmother, York:** *Because like new mums they should be able to get to a doctor shouldn’t they… in this day and age doctors are accessible but like the elderly, it’s even if they only live maybe half a mile from the doctors for an old person that half a mile can seem like ten miles to them. So for the elderly I think there should be a nurse for a couple of hours that could go out and give them their immunisation.*

Conversely, in London several Irish Travellers valued the Health Visitors who came to their sites to do vaccinations (especially during the time of a measles outbreak). They suggested that this is less stressful for the children, easier for those who travel as well as those who do not have a car to get to the GP practice; and that the Health Visitor takes more time discussing and giving the vaccination than occurs in the GP practice.

#### National strategies

##### Payment and incentives

A small minority of Traveller participants, all women, commented on the UK Government’s policy of free immunisation stating that if they had to pay for them, they could not afford them.

**BT108c, Irish Traveller, Adolescent girl with no children, Bristol:** *[Free immunisations] are a very good thing because there is a lot more than the Travelling community out there that would need these needles and they couldn’t afford it.*

Some Bristol Roma parents explained that in Romania you were required to pay for immunisations.

**BT201a, Romanian Roma, Mother, Bristol:** *It was difficult sometimes [in Romania], because some immunisation, they need to be paid, and have some immunisation free, but it’s very important here because it’s free.*

Finally, an Irish Traveller mother from London and an English Gypsy mother in York believed that childhood immunisations should be mandatory for attending school and people should be fined for not having them.

## Discussion

This is the first in-depth, qualitative study exploring Travellers’ views on childhood and selected adult immunisation from multiple communities and cities in the UK. The inclusion of diverse communities in four UK cities enabled us to identify differences and similarities in views *within* each community as well as drawing out meaningful comparisons across the six Traveller communities, both for gender and different vaccines. Use of the SEM [36] as an organising framework ensured that Travellers’ accounts were explored beyond their individual beliefs, experiences and behaviours, to include inter-personal, institutional, community and policy- related influences.

Participants represented a mix of family roles across generations and categories of immunisation status. We have no reason to believe that the communities in the study are markedly different to other Traveller communities of the same descent either in their acceptance of immunisation [12, 31, 33, 48] or their social contexts which impact on access to immunisation services [2, 49–51]. This and the rigour of the study design and conduct give confidence that findings are relevant to members of other Traveller communities of English, Irish, Romanian/Slovakian Roma and Scottish Showpeople descent are who are housed or who live on a caravan site. The findings cannot be fully extrapolated to Traveller families who relocate frequently as they may as they experience additional barriers to accessing immunisations.

Overall, it can be seen there were many common barriers and facilitators to immunisation uptake across all six communities which were similar to those found within the general population. Notably, the two Roma communities experienced additional barriers in terms of language and adapting to living in a new country. On the whole, men and women described similar barriers and facilitators. However, like the general population [52, 53], childhood immunisation was often regarded as an area in which women took more interest, and for which they took more responsibility, than men. Women were more likely than men to discuss discrimination, the importance of free vaccinations and low literacy barriers to uptake. Barriers and facilitators were identified across the five levels of the SEM [36]; although Travellers noticeably spoke less of policy level influences. These were more fully discussed within the interviews with Service Providers reported elsewhere [42]. The barriers and facilitators also reflected the two broad categories of factors which are considered to influence the uptake of childhood and adult immunisation [16–21], that is acceptance of vaccines and access to health services.

### Acceptance of vaccines

The majority of Traveller participants expressed positive attitudes towards immunisation. This was particularly evident amongst the Roma communities, followed closely by the Bristol English Gypsy/Irish Travellers and Glasgow Scottish Showpeople. A small minority in the four English-speaking communities were sceptical about immunisation in general and only three participants expressed views indicating outright rejection of vaccinations. Leask et al. [21] identify five parental positions towards immunisation with approximate estimates of the proportion of each group: the ‘unquestioning acceptor’ (30–40%), the ‘cautious acceptor’ (25–35%); the ‘hesitant’ (20–30%); the ‘late or selective vaccinator’ (2–27%); and the ‘refuser’ of all vaccines (<2%). These positions, based on the general population, were all evident amongst the Travellers we interviewed for childhood as well as adult vaccines; although participants discussed selective rather than late vaccination. Due to the qualitative nature of our study we cannot attribute proportions of participants to the five positions identified by Leask et al. Nevertheless, what is clear is that the distribution of these positions varied across Traveller communities. For example, most Roma participants would be classified as ‘unquestioning acceptors’ whereas the ‘selective vaccinator’ position was most evident within the English Gypsy and Irish Traveller communities. There are no existing studies investigating Travellers’ views on adult immunisation with which to compare our findings, however studies exploring Travellers’ (predominantly English Gypsy and Welsh Traveller communities) views on childhood immunisation also report mixed acceptance [12, 31, 48]. In those studies resistance to immunisations was associated with concerns about the potential side effects and a lack of belief in the value of vaccination.

Concerns about the safety of specific vaccines were primarily historic, predominantly held by older participants and focused on MMR and the whooping cough vaccine. This is not surprising given controversies over their safety in the late 1990s/2000s (MMR) and 1970s (whole cell whooping cough — which is no longer used in the UK). As in the general population the spread of information and misinformation can result in the ‘social amplification of risk’ that quickly influences perceptions and behaviours [54]. Indeed, this had occurred in the past for MMR in Bristol, York and London Traveller communities. However the data suggested that views have changed over time with the majority now accepting this combination vaccine which again, has parallels with the general population [55, 56]. This appeared to be associated with the current generation of Traveller parents having better knowledge about immunisation and opportunities to build trustful relationships with health professionals, thereby relying less on lay knowledge transmitted by family or community.

This study demonstrates that variable acceptance of the adult flu vaccination identified within the general population [57] extends to English-speaking Travellers communities. Some Traveller participants across all four English-speaking communities believed that it ‘caused flu’ and that in comparison to other immunisations is less important to have because of a perception that flu, in comparison to other vaccine preventable diseases, is relatively benign. The perception that vaccination can cause flu and concerns about side-effects have both been identified as deterrents to uptake in high risk older people and the general population [58].

### Access to health and immunisation services

Accounts from Travellers suggested that for the majority of English-speaking Traveller participants registering with a GP practice, being notified and reminded of immunisations (via letters, texts, telephone calls, face-to-face contact with health professionals) facilitated and promoted attendance for immunisations in primary care and schools. This may be related to the ‘settled’ nature of our sample who were housed or resident on authorised Traveller sites, many with established, long term relationships with GP practices and health professionals (although less so for the Roma families). Additionally, the use of reminders is one of the few interventions for which there is robust evidence of effectiveness in increasing vaccination uptake [59, 60]. Travellers’ apparent satisfaction may also reflect that the services are enhanced for some communities (particularly the Roma) which were described within the interviews with Service Providers [42]. The All Ireland Traveller Health Study Team [61] suggest that understanding access to health services is complex and whilst many of the Travellers in their study (and in other studies [4, 48, 62]) report using health services, these authors suggest it is the experience of that engagement which is important and too often is sub-optimal. In our study there were some Traveller participant accounts of frustrations with getting through on the telephone to make a GP appointment, the length of time, often several weeks, to get an appointment (including for immunisation), as well as having to wait lengthy periods in busy clinics to be seen by a health professional. This led to a minority preferring to use A&E or out of hours doctors as observed elsewhere [39, 61]. These criticisms of primary care similarly feature in national population surveys for England and Scotland [63] and are known to impact on people’s use of services.

With the exception of language barriers the Slovakian and Romanian Roma participants did not talk about the difficulties they faced accessing health services despite being relatively recent migrants to the UK (since the accession of their countries of origin to the European Union in 2004 and 2007). Inability to access health services effectively was a major need previously identified, particularly in Glasgow, in relation to the Roma community and so additional services had been put in place before this study was conducted. These were discussed at length in the Service Provider interviews [42]. We have no doubt that this impacted on the families’ views of the health services. Some Roma participants also reported a lack of discrimination in the delivery of services in the UK (by most service providers) — they had been used to high levels of discrimination back home. The lack of discrimination also appeared to influence their views on accessing health services. Our findings differ to other studies with Roma [5, 7] migrant [64] and minority ethnic communities [65] which report considerable barriers to accessing health services. There were other examples of difficulties in using immunisation services which were associated with broader, inter-related, socio-economic barriers that exist for Travellers and are known to impact on their access to health services more widely [1, 4, 38, 66]. We learnt from the interviews with Service Providers [42] that across the six Traveller communities, the Romanian Roma (and to a lesser extent Slovakian Roma) families appeared to live with the highest levels of socio-economic deprivation, which is well documented [5, 7, 30]. In contrast, the Glasgow Scottish Showpeople spoke much less about these challenges, again resonating with existing reports [2]. Showpeople generally run businesses, live in permanent homes in privately owned or leased yards and travel out to set up and run fairground attractions. Consistent with other studies [3, 31, 48] living on the roadside was perceived to make it difficult to register with a GP practice, receive Health Visitor services and to be informed of forthcoming immunisation appointments. Travelling less frequently, for example to summer fairs, was not seen as a barrier to immunisation by York English Gypsy or Glasgow Scottish Showpeople participants who said that they would return to a known health professional for a scheduled immunisation appointment.

Low literacy was also identified as a barrier, to understanding written information, invitation letters for immunisation as well as communicating with health professionals, especially GPs in consultations, even among the current generation of parents. There was widespread preference for simple written information with pictures and jargon-free spoken communication by health professionals. The Roma communities also spoke at length about the additional language barriers they face in accessing health services, relying heavily on interpreters and bi-lingual health workers which were often in short supply, particularly those speaking Roma (rather than Romanian or Slovakian). These literacy and language barriers affecting Travellers’ confidence in attending appointments and engaging in conversations with health professionals [8, 61, 66] for fear of feeling humiliated and shamed have been reported elsewhere.

Finally, trust in health professionals, particularly GPs, Health Visitors and bi-lingual Health Workers and relational continuity of care [65] were important factors influencing immunisation acceptance and experience. This is also the case for the general population [21 23] although is perhaps more pertinent to Travellers because of their history of not accessing preventive health services and of long-standing discrimination [39]. Many English-speaking Travellers spoke of attending the same GP practice and preferring to see the same health professional over many years. A small minority of Traveller participants from the English-speaking communities described a general lack of trust of health professionals relating to negative experiences, such as medical notes being lost or Health Visitors being perceived to be judgemental about Travellers’ culture. These experiences appeared to have damaged their relationships with health professionals and eroded trust. For some women these experiences were seen as examples of discrimination due to their Traveller status. These findings resonate strongly with other studies of Travellers’ experiences of health services [4, 39, 48, 61] in which health professionals who are culturally well-informed and respectful are highly valued [4, 39] and trust is developed through outreach workers mediating between health services and Travellers [61]. Also Travellers have attributed past medical errors to discriminatory lack of care based on being a Traveller [34]. Van Cleemput [39] argues that discrimination by health professionals is often associated with a lack of personal experience of working with Travellers, meaning that assumptions are made based on stereotypes. She, and other authors [2, 4, 8, 48, 50] identify a clear need for cultural awareness training for Service Providers.

## Conclusions

There were many common accounts of barriers and facilitators across the six communities, particularly across the four English-speaking Traveller communities. To some extent these mirrored views of the general population although some barriers were particular to Travellers which reflected access barriers to health services more generally, as well cultural beliefs which were an obstacle to vaccination uptake. Language barriers were of high concern to Roma participants and reduced their access to immunisations which were predominantly highly valued. Concerns about the moral correctness of adolescent girls having the HPV vaccine were common in the Bristol English Gypsy/Irish Traveller community, and presented the most specific cultural barrier to a vaccine.

The experience of many Travellers in this study, and the context through which they make health decisions, is changing. Historical beliefs held by health professionals regarding the barriers and facilitators to immunisation uptake may no longer be valid. This large qualitative study has identified key issues that should be considered when taking action to improve the uptake of immunisations in Traveller families and reduce the persistent inequalities in coverage.

## Additional file

Additional file 1: Thematic Framework. (DOCX 14 kb)

## References

[1] Aspinall P (2005). Report for health ASERT programme Wales.

[2] Cemlyn S, Greenfields M, Burnett S, Matthews Z, Whitwell C (2009). Inequalities experienced by gypsy and traveller communities: a review.

[3] Matthews Z (2008). The health of gypsies and travellers in the UK.

[4] Parry G, Van Cleemput P, Peters J, Walters S, Thomas K, Cooper C (2007). Health status of gypsies and travellers in England. J Epidemiol Community Health.

[5] Cook B, Wayne G, Valentine A, Lessios A, Yeh E (2013). Revisiting the evidence on health and health care disparities among the Roma: a systematic review 2003–2012. Int J Public Health.

[6] Aspinall P (2014). Hidden needs identifying key vulnerable groups in data collections: vulnerable migrants, gypsies and travellers, homeless people, and sex workers.

[7] Lane P, Spencer S, Jones A (2014). Gypsy, traveller and Roma: experts by experience.

[8] Jenkins L (2010). Preparation study of gypsy/traveller needs.

[9] Gordon M, Gorman D, Hashem S, Stewart D (1991). The health of travellers’ children in Northern Ireland. Public Health.

[10] Feder G, Vaclavik T, Streetly A (1993). Traveller gypsies and childhood immunization: a study in east London. Br J Gen Pract.

[11] Pahl J, Vaile M (1984). Health and health care among travellers. J Soc Policy.

[12] Moreton J (1992). HIB educating parents and professionals. Health Visit.

[13] Dar O, Gobin M, Hogarth S, Lane C, Ramsay M (2013). Mapping the gypsy traveller community in England: what we know about their health service provision and childhood immunization uptake. J Public Health.

[14] Maduma-Butshe M, McCarthy N (2012). The burden and impact of measles among gypsy-traveller communities, Thames valley, 2006–09. J Public Health.

[15] Department of Health (2010). Measles outbreaks in gypsy and travellers communities. Gateway reference number: 14614.

[16] Falagas M, Zarcadoula E (2008). Factors associated with suboptimal compliance to vaccinations in children in developed countries: a systematic review. Curr Med Res Opin.

[17] Brown K, Kroll J, Hudson M, Ramsay M, Green J, Long S (2010). Factors underlying parental decisions about combination childhood vaccinations including MMR: a systematic review. Vaccine.

[18] Tickner S, Leman P, Woodcock A (2006). Factors underlying suboptimal childhood immunisation. Vaccine.

[19] Mills E, Jadad A, Ross C, Wilson K (2005). Systematic review of qualitative studies exploring parental beliefs and attitudes toward childhood vaccination identifies common barriers to vaccination. J Clin Epidemiol.

[20] Yaqub O, Castle-Clarke S, Sevdalis N, Chataway J (2014). Attitudes to vaccination: a critical review. Soc Sci Med.

[21] Leask J, Kinnersley P, Jackson C, Cheater F, Bedford H, Rowles G (2012). Communicating with parents about vaccination: a framework for health professional. BMC Pediatr.

[22] Pearce A, Marshall H, Bedford H, Lynch J (2015). Barriers to childhood immunisation: findings from the longitudinal study of Australian children. Vaccine.

[23] European Centre for Disease Prevention and Control (2015). Rapid literature review on motivating hesitant population groups in Europe to vaccinate.

[24] Eilers R, Krabbe P, de Melker H (2014). Factors affecting the uptake of vaccination by the elderly in Western society. Prev Med.

[25] Wheelock A, Thomson A, Sevdalis N (2013). Social and psychological factors underlying adult vaccination behavior: lessons from seasonal influenza vaccination in the US and the UK. Expert Rev Vaccines.

[26] Ahmed F, Friedman C, Franks A, Latts L, Nugent E, France E (2004). Effect of the frequency of delivery of reminders and an influenza tool kit on increasing influenza vaccination rates among adults with high-risk conditions. Am J Manag Care.

[27] Bodeker B, Walter D, Reiter S, Wichman O (2014). Cross-sectional study on factors associated with influenza vaccine uptake and pertussis vaccination status among pregnant women in Germany. Vaccine.

[28] Wiley K, Massey P, Cooper S, Wood N, Ho J, Quinn H (2013). Uptake of influenza vaccine by pregnant women: a cross-sectional survey. Med J Aust.

[29] Samad L, Butler L, Peckham C, Bedford H (2006). Incomplete immunisation uptake in infancy: maternal reason. Vaccine.

[30] Sivic S, Huremovic A, Djerzic H (2013). Social exclusion as a determining health factor of the Roma population. Med Arch.

[31] Papadopoulos I, Lay M (2007). The health promotion needs and preferences of gypsy travellers in Wales. Diversity Health Social Care.

[32] Hawes D (1997). Gypsies, travellers and the health service.

[33] Boika R, Blackburn C, Spencer N, Rechel B (2009). Access to health care for Roma children in central and eastern Europe: findings from a qualitative study in Bulgaria. Int J Equity Health.

[34] Van Cleemput P (2007). Health-related beliefs and experiences of gypsies and travellers: a qualitative study. J Epidemiol Community Health.

[35] Jackson C, Bedford H, Condon L, Crocker A, Emslie C, Dyson L (2015). UNderstanding uptake of Immunisations in TravellIng aNd Gypsy communities (UNITING): protocol for an exploratory, qualitative study. BMJ Open.

[36] McLeroy K, Bibeau D, Steckler A, Glanz K (1988). An ecological perspective on health promotion programmes. Health Educ Q.

[37] HM Government. Race Relations Act. 1976. http://www.legislation.gov.uk/ukpga/1976/74. Accessed 11 Nov 2015.

[38] HM Government. Equality Act 2010. 2010. http://www.legislation.gov.uk/ukpga/2010/15/contents. Accessed 11 Nov 2015.

[39] Van Cleemput P (2010). Social exclusion of gypsies and travellers: health impact. J Res Nursing.

[40] Travellers Aid Trust. Grant Makers Fact Sheet on Gypsies and Travellers. http://www.travellersaidtrust.org/pdfs/grant%20makers%20fact%20sheet%2009.pdf. Accessed 11 Nov 2015.

[41] Scottish Traveller Education Programme. Travelling Communities in Scotland. 2013. http://www.step.education.ed.ac.uk/. Accessed 11 Nov 2015.

[42] Jackson C, Dyson L, Bedford H, Cheater FM, Condon L, Crocker A (2016). UNderstanding uptake of immunisations in TravellIng aNd gypsy communities (UNITING): a qualitative interview study. Health Technol Assess.

[43] Emmel N, Hughes K, Greenhalgh J, Sales A. Accessing Socially Excluded People — Trust and the Gatekeeper in the Researcher-Participant Relationship. Sociological Res Online. 2007;12.

[44] NHS. The routine immunisation schedule. 2016. https://www.gov.uk/government/publications/the-complete-routine-immunisation-schedule. Accessed 10 Mar 2017.

[45] Bryman A, Bryman A (1984). Analysing qualitative data.

[46] Ritchie J, Lewis J, McNaughton Nicholls C, Ormston R (2014). Qualitative research practice.

[47] Barnett-Page E, Thomas J (2009). Methods for the synthesis of qualitative research: a critical review. BMC Med Res Methodol.

[48] Twiselton I, Huntington F (2009). Health needs assessment: Cumbria gypsy travellers.

[49] Brown P, Scullion L, Martin P (2013). Migrant Roma in the United Kingdom. Population size and experiences of local authorities and partners. Final report.

[50] The Social Marketing Gateway (2013). Mapping the Roma community in Scotland. Final report.

[51] Office for National Statistics (2014). What does the 2011 census tell us about the characteristics of gypsy or Irish travellers in England and Wales?.

[52] Ranji U, Salganicoff A (2014). Balancing on shaking ground: women, work and family health.

[53] McMurray R, Cheater F, Weighall A, Nelson C, Schweiger M, Mukerjie S (2004). Managing controversy through consultation: a qualitative study of communication and trust around MMR vaccination. Br J Gen Pract.

[54] Larson H, Smith D, Paterson P, Cumming M, Eckersberger E, Freifeld C (2013). Measuring vaccine confidence: analysis of data obtained by a media surveillance system used to analyse public concerns about vaccines. Lancet Infectious Diseases.

[55] Brown K, Long S, Ramsay M, Hudson M, Green J, Vincent C (2012). UK parents’ decision-making about measles-mumps-rubella (MMR) vaccine 10 years after the MMR-autism controversy: a qualitative analysis. Vaccine.

[56] Public Health England (2015). Quarterly vaccination coverage statistics for children aged up to five years in the UK (COVER programme): January to March 2015.

[57] Nagata J, Hernández-Ramos I, Kurup A, Albrecht D, Vivas-Torrealba C, Franco-Paredes C (2013). Social determinants of health and seasonal influenza vaccination in adults ≥65 years: a systematic review of qualitative and quantitative data. BMC Public Health.

[58] Brondi L, Higgins M, Gorman D, McCormick D, McCallum A, Fisken S (2013). Review of the scientific literature on drivers and barriers of seasonal influenza coverage in the EU/EEA.

[59] Van Jacobson J, Szilagyi P (2005). Patient reminder and recall systems to improve immunization rates. Cochrane Database Syst Rev.

[60] Crocker-Buque T, Edelstein M, Mounier-Jack S (2016). Interventions to reduce inequalities in vaccine uptake in children and adolescents aged <19 years: a systematic review. J Epidemiol Community Health.

[61] All Ireland Traveller Health Study Team (2010). All Ireland traveller health study our geels.

[62] Mid Western Health Board (2009). Travellers’ perceptions and experiences of maternity & child health services.

[63] NHS England. GP Patient Survey 2014–15. 2015.

[64] Stagg H, Jones J, Bickler G, Abubakar I. Poor uptake of primary healthcare registration among recent entrants to the UK: a retrospective cohort study. BMJ Open. 2012;2. 10.1136/bmjopen-2012-001453 PMC4400681 22869094

[65] Burt J, Lloyd C, Campbell J, Roland M, Abel G (2015). Variations in GP–patient communication by ethnicity, age, and gender: evidence from a national primary care patient survey. Br J Gen Pract.

[66] Van Cleemput P, Bissell P, Harris J (2010). Pacesetters programme - gypsy, Roma and traveller core strand.

