# Supplementary material for: Needles, Jabs and Jags: a qualitative exploration of barriers and facilitators to child and adult immunisation uptake among Gypsies, Travellers and Roma
Source: BMC Public Health. 2017 Mar 14;17:254. doi: 10.1186/s12889-017-4178-y (PMC5348901; doi:10.1186/s12889-017-4178-y)
Supplement: Additional file 1: — Thematic Framework. (DOCX 14 kb) [file 12889_2017_4178_MOESM1_ESM.docx]

Additional File 1: **Thematic Framework**

Theme 1: Immunisation and health status

| ID | Age | Immunisation Status | Immunisation Status of Children | Immunisation Status of Grand Children | Terminology  (jabs, needles,  immunisations) | General Health Status (participant and family) | Literacy |
| --- | --- | --- | --- | --- | --- | --- | --- |

Theme 2: Indirect positive influences on immunisation attitudes/behaviour

| ID | First Hand | Second Hand (Relative) | Second Hand (Friend) | Second Hand (Health Professional) | Second Hand (Community) | Other |
| --- | --- | --- | --- | --- | --- | --- |

Theme 3: Indirect negative influences on immunisation attitudes/behaviour

| ID | First Hand | Second Hand (Relative) | Second Hand (Friend) | Second Hand (Health Professional) | Second Hand (Community) | Other |
| --- | --- | --- | --- | --- | --- | --- |

Theme 4: Knowledge of immunisation

| ID | Existing/Prior Knowledge | Source | Knowledge at Time of Decision | Source | Knowledge at Time of having Immunisation | Source |
| --- | --- | --- | --- | --- | --- | --- |

Theme 5: Reason(s) for general immunisation behaviour

| ID | Protection against Disease | Friends all have them | Advised by Health Professional | What my Parents did | Other |
| --- | --- | --- | --- | --- | --- |

Theme 6: Reason(s) for behaviour for a specific immunisation (if different to general)

| ID | Name of Immunisation | Protection against Disease | Friends all have them | Advised by Health Professional | What my Parents did | Other |
| --- | --- | --- | --- | --- | --- | --- |

Theme 7: Reason(s) for immunisation beliefs or behaviour for a family member

| ID | Name of Immunisation | Protection against Disease | Friends all have them | Advised by Health Professional | What my Parents did | Other |
| --- | --- | --- | --- | --- | --- | --- |

Theme 8: Prompt for attending for immunisation

| ID | Name of Immunisation | Letter from GP Practice | Previously booked appointment | Knew it was due from Record | Follow up for Missed Appointment | Attended with Someone else visiting GP Practice | Provided at School | Visit by outreach Health Professional | Other |
| --- | --- | --- | --- | --- | --- | --- | --- | --- | --- |

Theme 9: Family’, friends’ and others’ immunisation attitudes and behaviour

| ID | Family member | Partner | Friend | Health Professional | School | Community | Media | Inter-generational  Change |
| --- | --- | --- | --- | --- | --- | --- | --- | --- |

Theme 10: Barriers to immunisation(s)

| ID | Lack of awareness of need for Immunisation | Information difficult to understand | Lack of time from Health Professional | Hostility/Lack of respect towards Travellers | Negative image of Travellers in Media | Difficulty accessing Services as travelling | Difficulty of registration at GP practice when travelling | Daughter not at School | Mistrust of School-based Immunisation | Other |
| --- | --- | --- | --- | --- | --- | --- | --- | --- | --- | --- |

Theme 11: Ideas for interventions – information and culture

| ID | Increased acceptance /integration of Travellers in Community | Positive portrayal of Travellers within media | Verbal Information to promote Awareness | Simple Leaflets including pictures and appropriate text explaining benefits and side effects | Adverts on TV to promote specific immunisation campaigns | Role of older generations to promote Immunisations and support attendance for younger generations | Other |
| --- | --- | --- | --- | --- | --- | --- | --- |

Theme 12: Ideas for interventions – access to and quality of health care services

| ID | Increased access to Drop-in Centres | Outreach Services | Availability of GP as trusted Health Professional | Out of hours Services | Red Book | Invitation letter/text to attend Appointment | Reminder letter/text to attend Appointment | Follow up by Health Professionals Prof if miss Appointment | Being treated with respect by Health Professional | Easier temporary Registration | Other |
| --- | --- | --- | --- | --- | --- | --- | --- | --- | --- | --- | --- |
